# Draft Genome Sequence of a Porcine Commensal, *Rothia nasimurium*, Encoding a Nonribosomal Peptide Synthetase Predicted To Produce the Ionophore Antibiotic Valinomycin

**DOI:** 10.1128/genomeA.00453-17

**Published:** 2017-06-01

**Authors:** Rogier A. Gaiser, Marnix H. Medema, Michiel Kleerebezem, Peter van Baarlen, Jerry M. Wells

**Affiliations:** a Host-Microbe Interactomics Group, Animal Sciences Department, Wageningen University, Wageningen, The Netherlands; b Bioinformatics Group, Wageningen University, Wageningen, The Netherlands

## Abstract

We report the draft whole-genome sequence of *Rothia nasimurium* isolated from a porcine tonsil. The genome encodes a nonribosomal peptide synthetase predicted to produce valinomycin, a cyclic dodecadepsipeptide ionophore. Previously, valinomycin was known to be produced only by *Streptomyces* species and isolates belonging to the *Bacillus pumilus* group.

## GENOME ANNOUNCEMENT

Members of the* Rothia* genus are commonly found as commensal bacteria in oral and intestinal microbiomes of humans, pigs, and rodents (1–3). Here, we report the whole-genome sequence of a *Rothia nasimurium* isolate from palatine tonsils of healthy piglets that inhibits the growth of multiple strains and serotypes of the porcine pathogen *Streptococcus suis*, demonstrated using agar overlay and agar well diffusion methods. The genome sequence was determined to gain insight into the molecular and genetic basis underlying the antimicrobial potential of this isolate.

Genomic DNA was extracted from a culture of *Rothia nasimurium* isolate that we designated PT-32, grown in brain heart infusion broth (Oxoid, Basingstoke, UK), and sequenced at BaseClear (Leiden, The Netherlands) using an Illumina HiSeq 2500 system on a paired-end library with 125 cycles. FASTQ sequence files were generated using the Illumina Casava pipeline 1.8.3. Quality assessment was based on Illumina Chastity filtering, removal of reads containing adapters and/or PhiX control signal using a BaseClear in-house filtering protocol, and finally by using the FASTQC quality control tool version 0.10.0. CLC Genomics Workbench 8 was used for trimming of low-quality bases and assembly of reads into contigs. The optimal k-mer size was determined using KmerGenie (4). The contigs were linked and placed into scaffolds using the SSPACE Premium scaffolder 2.3 (5). GapFiller 1.10 (6) was used for automated closing of gapped regions within the scaffolds.

After filtering and quality assessment, 3,611,913 paired mapped reads were assembled into 45 scaffolds, creating a genome with a total size of 2,685,591 bp, having an average 170-fold coverage and an average G+C content of 57.98%. The NCBI Prokaryotic Genome Annotation Pipeline (7) predicted 2,215 protein-coding genes, 50 tRNA genes, and 3 rRNA genes. The 16S rRNA gene sequences of phylogenetic neighbors were found using the EzTaxon server (8), with strain PT-32 showing the highest sequence similarity (97.89%) to *R. nasimurium*.

antiSMASH 3.0 (9) was used for identification and annotation of secondary metabolite biosynthesis gene clusters. One nonribosomal peptide synthetase (NRPS) cluster was identified that codes for an NRPS assembly line organization identical to those of *vlm* from *Streptomyces* (10–14) and *ces* from *Bacillus* (15, 16). Based on the sequences and substrate specificity predictions of its adenylation domains, this NRPS cluster was predicted to synthesize a compound highly similar or identical to valinomycin, a cyclododecadepsipeptide ionophore with antibiotic activity (10, 11, 17, 18). This NRPS cluster is not present in any other publicly available *Rothia* genome.

This is the first report of a commensal bacterium from mammal-associated microbiota containing an NRPS cluster encoding a valinomycin-type nonribosomal peptide. The availability of this genome sequence will facilitate further studies on the prevalence and distribution of valinomycin-producing NRPS gene clusters and their ecological function.

### Accession number(s).

This whole-genome shotgun project has been deposited at DDBJ/ENA/GenBank under the accession no. LXWF00000000. The version described in this paper is version LXWF01000000.
